# Efficiency assessment and demand forecasting in China’s primary healthcare system: a comprehensive SBM-DDF-GML analysis

**DOI:** 10.3389/fpubh.2026.1726155

**Published:** 2026-02-02

**Authors:** Siye Huang, Yao Li, Daiqing Cao, Mengting Li, Muyao Zhou, Yueyan Zhao, Yang Yu, Liang Shen

**Affiliations:** 1 School of Management, Xuzhou Medical University, Xuzhou, Jiangsu, China; 2 School of Pharmacy, Xuzhou Medical University, Xuzhou, Jiangsu, China; 3 The Second School of Clinical Medicine, Xuzhou Medical University, Xuzhou, Jiangsu, China; 4 School of Medical Information and Engineering, Xuzhou Medical University, Xuzhou, Jiangsu, China

**Keywords:** demand forecasting, efficiency assessment, primary health care, SBM-DDF-GML model, unreasonable hospitalization

## Abstract

**Background:**

With the serious aging of society, the demand for high-quality primary healthcare has increased. However, inadequate primary healthcare capacity and suboptimal resource allocation are hindering its development. Consequently, establishing a comprehensive and scientific evaluation system for the efficiency of primary healthcare plays a crucial role.

**Methods:**

Based on the perspective of measuring the input–output efficiency of primary healthcare, this study identifies the potential number of unreasonable hospitalizations and proposes a comprehensive method combining the Slacks-Based Measure, Directional Distance Function, and Global Malmquist-Luenberger (SBM-DDF-GML) to conduct static and dynamic efficiency analyses of primary healthcare institutions across China from 2010 to 2022. Additionally, we forecast future primary healthcare demand using a random forest model.

**Results:**

From 2010 to 2022, the average SBM-DDF efficiency score of China’s primary healthcare institutions was 0.92. During this period, the eastern and western regions demonstrate higher average efficiency values compared to the central areas, which reflects regional imbalances. Furthermore, demand forecasts suggest that primary healthcare demand will rise by 2029. with projected outpatient visits reaching 1.07 billion.

**Conclusion:**

Given persistent regional disparities, strengthening regional collaboration, and optimizing resource distribution may provide valuable insights for policymakers. These measures will help bridge efficiency gaps and ensure equitable healthcare delivery.

## 1 Introduction

With the aging of the population, the demand for high-quality primary health care services is also increasing. According to the OECD (1), the proportion of the population over 80 years old is projected to rise from 4.8% in 2021 to 9.8% by 2050. However, it is important to note that the increase in medical demand may put pressure on the allocation of public resources and jeopardize the sustainability of the social health system (2). The government work report pointed out that China’s healthcare system is centered on public hospitals, which is facing multiple pressures such as unbalanced resources and aging (3). Additionally, China’s primary healthcare system mainly forms a service network through community health service centers, township health centers and village clinics. For a long time, community health services have been a weak link in the national healthcare system. However, primary health services, such as those provided in communities, have made significant contributions to the health of local residents (4). As a result, establishing a comprehensive and scientific evaluation system for the efficiency of primary healthcare plays a crucial role in helping policymakers allocate limited medical resources effectively (5).

The Chinese authorities place special emphasis on primary health care. In January 2025, the National Medical and Political Work Conference was held in Beijing. The conference emphasized the need to continue promoting the effective extension of high-quality medical resources in two key directions: grassroots foundations and short-board specialties. This initiative aims to improve primary healthcare efficiency and promote balanced regional development. According to data from the Statistics Information Center, as of November 2024, primary medical institutions (excluding clinics, infirmaries, and village clinics) recorded 2.4 billion patient visits, representing a month-on-month growth of 3.4%. Among these visits, community health service centers accounted for 1.0 billion visits, a 5.8% increase from the previous year; township health centers accounted for 1.1 billion visits, with a year-on-year increase of 0.7% (6). The statistical time span is once a month, covering all provinces in the geographical area except Hong Kong, Macao and Taiwan, and the total rural population is 745,761,148 according to the latest census.

However, it is worth noting that whether the primary medical needs can be effectively met is closely related to the efficiency of medical resource allocation in different regions. Regions with high efficiency are more likely to meet the needs of patients. Therefore, scientific evaluation of primary health care efficiency is very important. In various fields of health care, Data Envelopment Analysis (DEA) model is widely used to evaluate relative efficiency (7–9). And the concept of “undesired output” already existed in previous publications (10–14), but the challenge was in how to scientifically measure it. Therefore, the first motivation of this study is to scientifically evaluate the efficiency of primary medical resource allocation by introducing an undesired output—unreasonable hospitalization. This approach aims to avoid the waste of medical resources caused by the blind pursuit of economic benefits by medical institutions. In addition, this study integrates the allocation efficiency of primary care from both dynamic and static perspectives. This is different from the work of Sun et al. (15), which evaluates the allocation efficiency of China’s medical resources in a specific year from a static perspective only.

Given the shortage of medical resources in China and the increasing complexity of health conditions, accurately predicting patient visits is particularly important and critical for the planning and allocation of medical resources (16). However, existing studies predominantly focus on evaluating the efficiency of healthcare resource allocation (17, 18), yet they largely neglect the prediction of medical demand. This challenges policymakers in rationalizing healthcare resource allocation. To address this, this paper proposes a model for predicting primary care needs, aiming to forecast future patient visits and support rational resource allocation.

In summary, this study identifies potential number of unreasonable hospitalizations by examining input–output efficiency and incorporates undesirable outputs into the evaluation framework to assess the efficiency of primary care resource allocation more scientifically. Building on the traditional Data Envelopment Analysis (DEA), this paper integrates a comprehensive approach that combines the Slacks-Based Measure (SBM), Directional Distance Function (DDF), and Global Malmquist–Luenberger (GML) to analyze the static and dynamic efficiency of primary medical institutions in China from 2010 to 2022. Given that existing research rarely addresses medical demand forecasting following efficiency evaluations, we also develop a primary care demand forecasting model. The aim is to offer practical insights and a scientific basis for improving the efficient allocation of primary care resources in other countries or regions.

## 2 Literature review

Primary medical and health institutions play a crucial role in disease prevention and health maintenance, so measuring the efficiency of resource allocation in these institutions has garnered increasing attention (19, 20). This section focuses on practical application of methods for assessing efficiency and forecasting demand within China’s primary health care system. Through scientific and critical analysis, it provides practical guidelines for the rational allocation of medical resources by the government.

### 2.1 Assessment of healthcare efficiency

In the field of health care, efficiency of medical resource allocation reflects the ratio of output to input of medical resources (15). To assess the efficiency of medical resource allocation, two methods are commonly used: parametric methods (such as stochastic frontier analysis, or SFA) and non-parametric methods (such as data envelopment analysis, or DEA) (20). Aigner et al. (21), Meeusen and Van Den Broeck (22) independently proposed Stochastic Frontier Analysis (SFA). This method is a mixed-effects model that evaluates the efficiency of a technique by breaking down the error term. For example, Cavalieri et al. (23) used SFA to test spatial dependence and heterogeneity of technical efficiency in Italian hospitals.

However, the stochastic frontier production function is suitable for datasets with large sample sizes and high quality, but it has certain limitations when applied to small samples, such as those found in primary medical and health institutions. As a result, the traditional data envelopment analysis (DEA) method is often used instead. In a thematic review of studies on the efficiency of health systems in Organization for Economic Co-operation and Development (OECD) countries, Alatawi et al. (24) found that DEA was used in 64% of studies to evaluate efficiency (25). DEA was originally proposed by Charnes and Cooper (26) to determine the relative efficiency of decision-making units through their input–output ratios. Subsequently, traditional DEA models, such as the Charnes-Cooper-Rhodes (CCR) and Banker-Charnes-Cooper (BCC), have been widely favored by scholars in the field of medical and health care (27–29).

Traditional DEA models have limitations, as they often ignore undesired outputs and treat them as normal outputs. To address this issue and establish a more realistic model, Tone (30) proposed an SBM model based on the slack variable metric. This model was further extended by Tone (31) to distinguish between desired and undesired outputs. Currently, it has been widely applied in various fields, including environmental science and healthcare (32–34). In addition, Fukuyama and Weber (35) innovatively integrated the SBM model with the DDF model, thereby significantly enhancing the accuracy of efficiency measurements. To avoid evaluation bias caused by slack variables, Tone (36) improved the model by directly addressing the slack problem through proportional adjustments of inputs and outputs based on these variables.

However, the SBM-DDF model is limited to evaluating efficiency in a static state and cannot meet the needs of dynamic analysis. To address this, Chung and Fare (37) combined the directional distance function (DDF) with the Malmquist productivity index to propose the Malmquist-Luenberger (ML) index. This index enables the analysis of dynamic efficiency changes over time. In particular, since Oh (38) incorporated undesirable outputs into efficiency assessments, the Global Malmquist-Luenberger (GML) index has gained wider application (39, 40). The system of evaluating the relative efficiency of data envelopment is becoming more and more mature and has been widely used (41–43). This paper aims to build on existing research to integrate an SBM-DDF-GML model for a comprehensive and scientific evaluation of the allocation efficiency of primary care resources. This work holds significant research value.

### 2.2 Healthcare demand forecasting

In recent years, forecasting medical and health needs has garnered increasing attention. This includes predicting the number of patient visits, demand for inpatient beds, and the demand for medical services (16, 44–47). With the rapid advancement of artificial intelligence, machine learning is being increasingly adopted across various fields. Applied five machine learning methods—Logistic Regression (LR), LR with LASSO regularization, Support Vector Machine (SVM), Random Forest (RF), and Extreme Gradient Boosting (XGBoost)—to compare their effectiveness in predicting healthcare service needs. Their study demonstrated that the RF model is one of the best performers. The random forest prediction method is a non-parametric approach in which each decision tree predicts a numerical value for the observations. The final prediction is then determined by majority voting or averaging (48). Hu and Szymczak (49) found that random forests perform well for predicting longitudinal data in the medical field. This paper aims to develop a random forest model to predict the number of primary care visits over the next few years, thereby providing a reference for the rational allocation of medical resources.

## 3 Materials and methods

**Figure 1:**
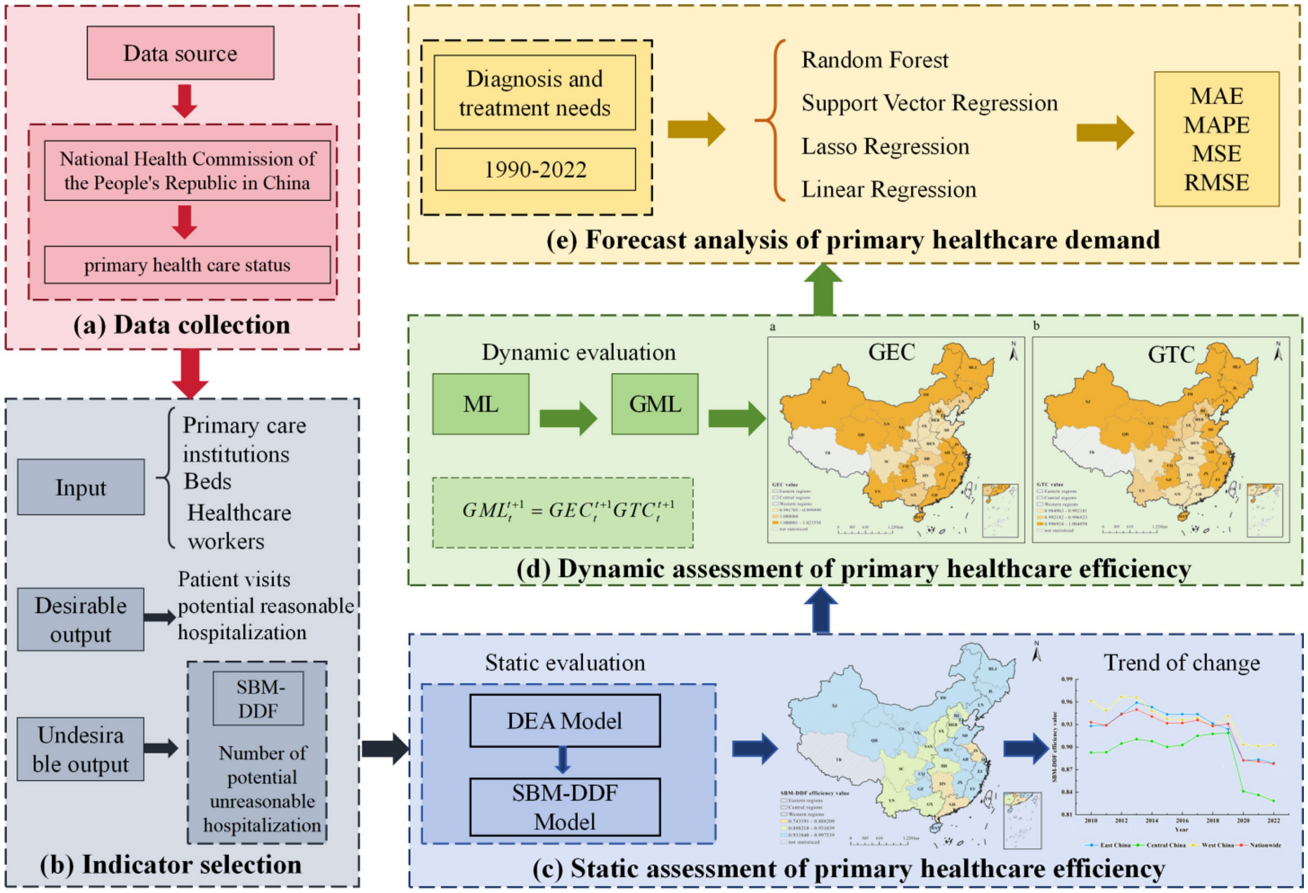
Framework for assessment and demand forecasting in China’s primary healthcare system.

Compared to the comprehensive SBM-DDF-GML model, traditional data envelopment analysis (DEA) has certain limitations. First, the radial DEA model ignores slack variables, allowing only proportional adjustments to all inputs or outputs of inefficient decision-making units ( DMUs ), which leads to biased efficiency estimates. Between 2010 and 2022, the Banker-Charnes-Cooper (BCC) model assessed primary healthcare efficiency across 30 Chinese provinces and municipalities, revealing an inefficiency rate as high as 50%. Second, traditional DEA often disregards undesirable outputs, which does not fully reflect reality. Therefore, this study adopts a macro input–output perspective, incorporating the potential number of unreasonable hospitalizations as undesirable output into the model. Building upon the work of Tone (31) and Fukuyama & Weber (35), we combine the Direction Distance Function (DDF) with Slacks-Based Measure (SBM) to achieve efficiency evaluation that includes undesirable outputs. Third, as the SBM-DDF model is limited to static efficiency assessment, this study employs the Global Malmquist-Ljungberg (GML) index proposed by Oh (38) to analyze the dynamic trends in China’s primary healthcare efficiency from 2010 to 2022. This index decomposes changes in total factor productivity into efficiency changes and technological changes, thereby revealing the dynamic characteristics of productivity growth (Figure 1).

### 3.1 Research method

#### 3.1.1 Static assessment: the SBM-DDF model

Fukuyama and Weber (35) proposed the SBM-DDF model to construct an improved relaxation measure directional distance function. On the basis of data standardization, MATLAB software is employed to construct the SBM-DDF model, which is used to evaluate the allocation efficiency of primary medical care in China over the period from 2010 to 2022. Assuming that among the 30 provinces in China, the first *k* provincial primary medical institution is one $\mathrm{DM}U_{k}$ , and each $\mathrm{DM}U_{k}$ has input factors $x=(x_{1},x_{2,\cdots }x_{n})\in R_{N}^{+}$ , the formula of the global SBM direction distance function is as follows:


$$\begin{array}{l} {\vec{S}}_{V}^{G}(x^{t,k^{\prime }},y^{t,k^{\prime }},b^{t,k^{\prime }},g^{x},g^{y},g^{b})\\ =\max_{s^{x},s^{y},s^{b}}\frac{\frac{1}{N}\sum_{n=1}^{N}\frac{s_{n}^{x}}{g_{n}^{x}}+\frac{1}{M+1}(\sum_{m=1}^{M}\frac{s_{m}^{y}}{g_{m}^{y}}+\sum_{i=1}^{I}\frac{s_{i}^{b}}{g_{i}^{b}})}{2} \end{array}$$

$$s.t.\{\begin{array}{l} \sum_{t=1}^{T}\sum_{k=1}^{K}w_{k}^{t}x_{\mathrm{kn}}^{t}+s_{n}^{x}=x_{k^{,}n}^{t},\forall_{n}\\ \sum_{t=1}^{T}\sum_{k=1}^{K}w_{k}^{t}y_{\mathrm{km}}^{t}+s_{m}^{x}=y_{k^{,}m}^{t},\forall_{m}\\ \sum_{t=1}^{T}\sum_{k=1}^{K}w_{k}^{t}y_{\mathrm{ki}}^{t}+s_{i}^{b}=b_{k^{,}i}^{t},\forall_{i}\\ \sum_{k=1}^{T}w_{k}^{t}=1,w_{k}^{t}\geq 0,\forall_{k}\\ s_{m}^{y}\geq 0,\forall_{m}\\ s_{i}^{b}\geq 0,\forall_{i} \end{array}$$

In the formula, $\left(g^{x},g^{y},g^{b}\right)$ represents the decrease in primary care input, the increase in expected output and the decrease in undesired output. $\left(s^{x},s^{y},s^{b}\right)$ are known as relaxation variables, representing input redundancy, insufficient expected output, and excessive undesired output, respectively. Additionally, based on the assumption of variable returns to scale, the $\sum_{j=1}^{n}\lambda_{j}=1$ equation constraints are added to make the model more reasonable.

#### 3.1.2 Dynamic assessment: the GML index

Following Oh’s (38) approach, this paper introduces the GML index in conjunction with the SBM-DDF model. This integration effectively resolves the issue of unsolvable linear programming under variable returns to scale and offers the significant advantages of transitivity and multiplicativity. The GML function is as follows:

First, the GML productivity index for the period from t to t+1 is expressed as:


$$\mathrm{GM}L_{t}^{t+1}=\frac{1+\vec{S_{V}^{G}}(x^{t};y^{t};b^{t};g^{x};g^{y};g^{b})}{1+\vec{S_{V}^{G}}(x^{t+1};y^{t+1};b^{t+1};g^{x};g^{y};g^{b})}$$

Secondly, the GML index can be further decomposed into the product of the Global Efficiency Change (GEC) and the Global Technological Change (GTC), which is expressed as follows:


$$\mathrm{GM}L_{t}^{t+1}=\mathrm{GE}C_{t}^{t+1}\mathrm{GT}C_{t}^{t+1}$$

$$\mathrm{GE}C_{t}^{t+1}=\frac{1+\vec{S_{V}^{G}}(x^{t};y^{t};b^{t};g^{x};g^{y};g^{b})}{1+\vec{S_{V}^{G}}(x^{t+1};y^{t+1};b^{t+1};g^{x};g^{y};g^{b})}$$

$$\mathrm{GT}C_{t}^{t+1}=\frac{\left[1+\vec{S_{V}^{G}}(x^{t},y^{t},b^{t};g^{x},g^{y},g^{b})][1+\vec{S_{V}^{t}}(x^{t+1},y^{t+1},b^{t+1};g^{x},g^{y},g^{b})\right]}{\left[1+\vec{S_{V}^{t}}(x^{t},y^{t},b^{t};g^{x},g^{y},g^{b})][1+\vec{S_{V}^{G}}(x^{t+1},y^{t+1},b^{t+1};g^{x},g^{y},g^{b})\right]}$$

Among them, GEC mainly refers to the improvement of the management system and medical resource allocation mode of primary medical and health institutions, and GTC mainly refers to the improvement of medical outpatient technology and clinical surgical skills. If the measured index is greater than 1, it indicates an improvement in total factor productivity, technical efficiency, and technological progress compared to the previous period of primary medical efficiency, showing a positive growth trend. Conversely, if the index is less than 1, it suggests a decline or downward trend. If the index equals 1, it signifies that the efficiency remains stable.

#### 3.1.3 Forecast analysis: primary healthcare demand

With growing attention to healthcare demand forecasting, this study investigates future trends in township primary health center (PHC) demand over the next few years to optimize primary healthcare resource allocation. Common prediction methods include Random Forests (RF), Support Vector Regression (SVR), and correlation analysis and so on (44–47). Research has shown that Random Forest excels at predicting longitudinal medical data (49). To achieve scientific forecasting, we compare Random Forests, SVR, Lasso Regression, and Linear Regression, selecting the most effective model.

To ensure a fair comparison, all models were trained on the same 33-year time series of annual patient visits to township-level primary health centers (PHCs), extracted from the China Health Statistical Yearbook (National Health Commission, 1990–2022). Given the inherent temporal dependence and continuity of time series data, this study employs single time steps as input features to construct a direct multi-step model, addressing the multi-period forecasting needs of township-level primary health centers. For dataset partitioning, we selected 29 years (1990–2018) from the training set to provide sufficient temporal patterns and periodic samples, while using the remaining 4 years (2019–2022) as the test set to evaluate model generalization. The overall split maintains a reasonable 9:1 ratio. Notably, constrained by the limited dataset size, sequential partitioning was adopted instead of cross-validation to ensure adequate training samples. Since the validation method demonstrates limitations compared to the robust time series cross-validation (TSCV), the predicted healthcare demand values carry inherent uncertainty and should be considered preliminary references.

This study selected four metrics: Mean Absolute Error (MAE), Mean Absolute Percentage Error (MAPE), Mean Squared Error (MSE), and Root Mean Squared Error (RMSE) to measure the regression prediction results. These metrics help assess the accuracy of the predicted values against the true values. The formulas for MAPE and RMSE are provided below.

MAPE: This metric measures the average percentage error between the predicted and actual values. It represents the mean of the absolute percentage differences between the predicted and actual values, calculated as follows:


$$\text{MAPE}=\frac{1}{n}\sum_{i=1}^{n}|\frac{y_{i}-{\hat{y}}_{i}}{y_{i}}|\times 100\%$$

RMSE: This metric is the square root of the Mean Squared Error (MSE) and quantifies the average magnitude of the deviation between the predicted values and the true values. The formula is as follows:


$$\text{RMSE}=\sqrt{\frac{1}{n}\sum_{i=1}^{1}{\left(y_{i}-{\hat{y}}_{i}\right)}^{2}}$$

Here, $y_{i}$ represents the true value, ${\hat{y}}_{i}$ represents the predicted value, and *n* is the total number of data points.

### 3.2 Data source and indicator selection

#### 3.2.1 Data source

The dataset for this study was obtained from the China Health Statistical Yearbook on the official website of the National Health Commission of the People’s Republic of China (50). Considering the availability and reliability of the data, this paper draws on the research ideas of Sun et al. (15), which Hong Kong, Macau, Taiwan and Tibet are not included in the study sample. That is, it mainly focuses on the primary medical institutions’ activities within the scope of 30 provinces, autonomous regions and municipalities from 2010 to 2022. In order to better grasp the collected data, the descriptive statistical results are detailed in Table 1.

**Table 1:** Descriptive statistics of relevant data.

Variables	Sample size	Mean	Standard deviation	Minimum	Maximum	Median
Primary care institutions	390	939963.39	26441.32	901,709	979,768	933,024
Beds	390	1475431.39	179432.76	1,192,242	1,751,081	1,441,940
Patient visits (10 thousand)	390	422475.32	25992.17	361,156	453,088	432,431
Hospital admissions (10 thousand)	390	4047.42	294.99	3,593	4,453	4,095

#### 3.2.2 Indicator selection

Selecting appropriate input and output indicators is crucial in evaluating the efficiency of primary healthcare resource allocation. The input–output selection needs to cover the necessary medical resources, and the outputs need to be consistent with the management objectives of the decision-making unit (27). Based on the literature on efficiency assessment in the healthcare industry (18, 51–54), we found that capital and labor were selected as input indicators to measure efficiency, i.e., beds, medical facilities can represent capital, and healthcare workers can represent labor. Since many of these factors are either difficult to quantify or fall outside the scope of direct medical and health control, this paper focuses on using the number of primary care institutions, beds, and healthcare workers as input indicators to assess the efficiency of primary medical care.

Imani et al. (55) found that output indicators used to analyze hospital efficiency often include outcome variables related to hospital activity and quality. In the existing literature, the number of patients and the number of hospital admissions have frequently been used as output indicators (17, 52, 56, 57). Since diagnosis and hospitalization are the core activities of medical institutions, the number of visits and hospitalizations is very important for the selection of indicators. However, it should be noted that the output evaluation index system can be divided into desired and undesired outputs. Undesirable output is a byproduct of ideal output (10). Zeng et al. (18) found that burdensome health care services are often undesirable outcomes of primary health care (PHC) systems. In view of the challenges of primiary health care in China (58), this paper further explores the situation of “potential number of unreasonable hospitalizations” in primary health care institutions as an undesirable output.

On the issue of identifying “potential number of unreasonable hospitalizations,” this paper draws on the research ideas of Liu (59) so we consider primary medical and health institutions in various regions as a tested input–output system. Using a single-output objective and the SBM-DDF framework, we assess how efficiently primary-care institutions allocate resources. From an input–output perspective, the decision-making unit’s efficiency value below 1 indicates it lies outside the efficiency frontier, signaling possible output shortfalls and input waste (59). Under the current model framework, the research assumes homogeneous outputs and adopts an output-oriented approach. By identifying macro-level efficiency losses through low efficiency values, the study decomposes the “potential number of unreasonable hospitalizations.” Furthermore, to further investigate the severity of the potential unreasonable volume of hospitalizations, this study acknowledges that healthcare resource allocation, medical equipment conditions, and service content may vary across regions. Therefore, a single indicator cannot adequately measure the severity of the potential unreasonable volume of hospitalizations. However, by treating primary healthcare institutions as an input–output system and evaluating the number of hospitalization from a macro perspective, we can both identify potential number of unreasonable hospitalization and eliminate the influence of input–output difference caused by misjudgment. Here, we quantify this through a “ $\rho_{1}-\rho_{2}$ ”: $\rho_{1}$ represents the input–output efficiency calculated without considering the number of hospitalization as an undesirable output, while $\rho_{2}$ reflects the efficiency when the number of hospitalization is included. A positive difference $\rho_{1}-\rho_{2}>0$ indicates that incorporating hospitalization frequency reduces efficiency, suggesting it may negatively impact healthcare operations. Conversely, a negative difference $\rho_{1}-\rho_{2}<0$ suggests that hospitalization, as a core medical service function, positively contributes to primary healthcare institutions’ operations. Building on this, we define a set of indicators to measure primary-care efficiency, laying the groundwork for the full efficiency analysis that follows. The detailed input–output indicator description is given in Table 2.

**Table 2:** Input–output indicator description.

Categories	Variables	Description
Input	Number of primary care institutions	The total count of primary medical facilities or organizations within a specific region or system.
	Number of beds	The total quantity of beds available in a primary hospital or across multiple primary medical facilities.
	Number of healthcare workers	The total count of individuals employed in the primary healthcare sector to provide medical and health-related services.
Desirable output	Number of patient visits	The total number of times patients seek primary medical care or consultation at a primary healthcare facility during a specific period.
	Number of potential reasonable hospitalization	The number of patients potentially meeting reasonable hospitalization criteria under specific conditions, as assessed by medical necessity.
Undesirable output	Number of potential unreasonable hospitalization	The number of potentially unnecessary hospitalizations estimated by the model.

## 4 Results

This study employs a two-stage analytical approach based on the SBM-DDF framework to comprehensively evaluate the efficiency of primary healthcare institutions in China. First, a benchmark efficiency assessment is conducted to identify macro-level inefficiencies and decompose total hospitalizations into “reasonable” and “potentially unreasonable” components. Subsequently, based on the decomposition results, a refined evaluation system is constructed by incorporating “the number of potentially unreasonable hospitalizations” as an undesirable output. This system integrates the SBM-DDF model with the Global Malmquist-Luenberger (GML) index to form a comprehensive static and dynamic efficiency analysis. The following sections present the specific results of this analytical process.

It is important to note that although both Section 4.1 and Section 4.2 utilize the same SBM-DDF framework, their methodological focuses differ fundamentally. Section 4.1 treats primary healthcare institutions as an aggregated input–output system. It first quantifies the potential scale of unreasonable hospitalizations by analyzing macro-level efficiency losses, and then employs a “decline ratio” to assess the efficiency difference before and after treating hospitalizations as an undesirable output, thereby measuring the severity of potentially unreasonable hospitalization practices. Building on this foundation, Section 4.2 utilizes the decomposed volumes of reasonable and unreasonable hospitalizations to construct a scientifically rigorous indicator system for static comprehensive analysis. This, in turn, integrates with the subsequent Section 4.3 to form a combined static-dynamic analytical approach. Therefore, the use of data from Table 3 for the main analysis and data from Table 4 for the calculation of “potentially unreasonable hospitalizations” is not contradictory but rather complementary. The two datasets represent successive and interdependent stages within the same analytical framework.

**Table 3:** Efficiency value of primary healthcare in China from 2010 to 2022.

Province	2010	2011	2012	2013	2014	2015	2016	2017	2018	2019	2020	2021	2022	Average
Beijing	0.99	0.99	0.99	1.00	1.00	1.00	1.00	1.00	1.00	1.00	0.98	0.99	0.99	**0.99**
Tianjin	1.00	1.00	1.00	1.00	1.00	1.00	1.00	1.00	1.00	1.00	0.99	0.99	0.99	**1.00**
Hebei	0.75	0.76	0.78	0.80	0.79	0.79	0.79	0.75	0.71	0.70	0.69	0.68	0.66	**0.74**
Shanxi	0.85	0.86	0.86	0.86	0.86	0.86	0.86	0.86	0.85	0.85	0.85	0.85	0.86	0.86
Inner Mongolia	0.94	0.93	0.93	0.93	0.93	0.93	0.93	0.92	0.92	0.92	0.91	0.91	0.91	0.92
Liaoning	0.89	0.89	0.89	0.90	0.90	0.90	0.89	0.89	0.88	0.89	0.88	0.87	0.88	0.89
Jilin	0.94	0.94	0.94	0.94	0.94	0.94	0.94	0.94	0.93	0.93	0.91	0.91	0.91	0.93
Heilongjiang	0.93	0.93	0.93	0.94	0.93	0.93	0.93	0.93	0.92	0.91	0.91	0.91	0.91	0.92
Shanghai	1.00	1.00	1.00	1.00	1.00	1.00	1.00	1.00	1.00	1.00	0.98	0.98	0.97	0.99
Jiangsu	0.91	0.91	0.93	0.95	0.96	0.97	0.96	1.00	0.96	0.94	0.84	0.84	0.80	0.92
Zhejiang	0.96	0.96	0.97	0.97	0.98	0.98	0.98	0.99	0.99	1.00	0.97	0.98	1.00	0.98
Anhui	0.93	0.92	0.95	0.97	0.97	0.95	0.95	0.95	0.93	0.92	0.89	0.88	0.86	0.93
Fujian	0.95	0.95	0.95	0.94	0.94	0.93	0.93	0.93	0.92	0.92	0.92	0.92	0.91	0.93
Jiangxi	1.00	1.00	0.94	0.95	0.93	0.92	0.92	0.93	0.92	0.91	0.88	0.87	0.86	0.93
Shandong	0.79	0.81	1.00	1.00	0.92	0.82	0.83	0.83	0.80	0.73	0.64	0.66	0.68	0.81
Henan	0.81	0.81	0.87	0.83	0.87	0.86	0.87	0.90	0.85	0.83	0.71	0.68	0.63	0.81
Hubei	0.89	0.89	0.93	0.94	0.93	0.92	0.93	1.00	0.93	1.00	0.81	0.82	0.81	0.91
Hunan	0.79	0.80	0.82	0.85	0.82	0.81	0.83	0.82	1.00	1.00	0.77	0.77	0.79	0.84
Guangdong	0.97	0.95	0.97	1.00	1.00	0.99	1.00	1.00	0.99	1.00	0.83	0.83	0.81	0.95
Guangxi	0.97	0.93	0.96	1.00	0.95	0.93	0.92	0.90	0.89	0.90	0.86	0.85	0.84	0.92
Hainan	1.00	1.00	0.90	1.00	1.00	1.00	1.00	0.99	0.99	0.99	0.99	0.98	0.98	0.99
Chongqing	0.98	0.97	1.00	1.00	0.99	0.97	0.97	1.00	0.97	1.00	0.96	0.97	0.95	0.98
Sichuan	0.93	0.87	1.00	0.94	0.87	0.83	0.85	0.90	0.85	1.00	0.74	0.73	0.76	0.87
Guizhou	1.00	0.98	1.00	0.99	0.94	0.92	0.91	0.90	0.90	0.90	0.87	0.88	0.89	0.93
Yunnan	0.97	0.96	0.97	0.97	0.96	0.95	0.95	0.94	0.93	0.92	0.90	0.91	0.91	0.94
Shaanxi	0.90	0.89	0.89	0.90	0.90	0.89	0.89	0.89	0.88	0.87	0.86	0.86	0.86	0.88
Gansu	0.94	0.94	0.93	0.94	0.94	0.94	0.94	0.93	0.93	0.93	0.92	0.91	0.91	0.93
Qinghai	1.00	1.00	0.99	0.99	0.99	0.99	0.99	0.99	0.99	0.99	0.99	0.99	0.99	0.99
Ningxia	1.00	1.00	1.00	1.00	1.00	1.00	1.00	1.00	1.00	1.00	0.99	0.99	0.99	**1.00**
Xinjiang	0.97	0.96	0.96	0.96	0.96	0.96	0.96	0.95	0.94	0.94	0.93	0.92	0.93	0.95
Average	0.93	0.93	0.94	0.95	0.94	0.93	0.93	0.93	0.93	0.93	0.88	0.88	0.87	**0.92**

**Table 4:** Input–output efficiency and its mean value (SBM-DDF) from 2010 to 2022.

Province	2010	2011	2012	2013	2014	2015	2016	2017	2018	2019	2020	2021	2022	Average ( $\bar{\rho_{1}^{*}}$ )
Beijing	0.99	0.99	0.99	1.00	1.00	1.00	1.00	1.00	1.00	1.00	0.98	0.99	0.99	0.99
Tianjin	1.00	1.00	1.00	1.00	1.00	1.00	1.00	1.00	1.00	0.99	0.99	0.99	0.98	1.00
Hebei	0.84	0.84	0.85	0.87	0.86	0.86	0.86	0.84	0.81	0.79	0.76	0.73	0.71	0.82
Shanxi	0.86	0.86	0.87	0.87	0.87	0.86	0.86	0.86	0.85	0.85	0.84	0.83	0.83	0.85
Inner Mongolia	0.94	0.93	0.93	0.93	0.93	0.93	0.93	0.92	0.91	0.91	0.90	0.90	0.89	0.92
Liaoning	0.90	0.90	0.90	0.90	0.91	0.90	0.90	0.90	0.89	0.88	0.87	0.86	0.86	0.89
Jilin	0.94	0.94	0.94	0.93	0.93	0.93	0.93	0.93	0.92	0.92	0.90	0.90	0.90	0.92
Heilongjiang	0.94	0.92	0.93	0.94	0.93	0.93	0.93	0.93	0.92	0.90	0.90	0.89	0.89	0.92
Shanghai	1.00	1.00	1.00	1.00	1.00	1.00	1.00	1.00	1.00	1.00	0.98	0.98	0.97	0.99
Jiangsu	0.94	0.94	0.96	0.97	0.98	0.98	0.98	1.00	0.98	0.97	0.91	0.91	0.88	0.95
Zhejiang	0.96	0.96	0.97	0.97	0.98	0.98	0.99	0.99	0.99	1.00	0.97	0.98	1.00	0.98
Anhui	0.95	0.94	0.97	0.98	0.98	0.97	0.97	0.97	0.96	0.95	0.92	0.90	0.89	0.95
Fujian	0.96	0.96	0.96	0.95	0.95	0.94	0.94	0.93	0.93	0.93	0.92	0.92	0.91	0.94
Jiangxi	1.00	1.00	0.97	0.97	0.96	0.95	0.95	0.96	0.96	0.94	0.91	0.91	0.90	0.95
Shandong	0.90	0.91	1.00	1.00	0.97	0.92	0.93	0.93	0.91	0.87	0.82	0.83	0.85	0.91
Henan	0.92	0.91	0.94	0.92	0.94	0.94	0.94	0.96	0.94	0.93	0.87	0.85	0.83	0.91
Hubei	0.93	0.93	0.96	0.97	0.97	0.96	0.97	1.00	0.97	1.00	0.90	0.91	0.90	0.95
Hunan	0.89	0.91	0.92	0.94	0.93	0.92	0.93	0.93	1.00	1.00	0.91	0.91	0.91	0.93
Guangdong	0.98	0.97	0.98	1.00	1.00	0.99	1.00	1.00	0.99	1.00	0.89	0.89	0.88	0.97
Guangxi	0.98	0.96	0.98	1.00	0.98	0.96	0.95	0.95	0.94	0.95	0.92	0.92	0.92	0.95
Hainan	1.00	1.00	0.91	0.99	1.00	0.99	0.99	0.99	0.99	0.99	0.98	0.98	0.98	0.98
Chongqing	0.98	0.98	1.00	1.00	0.99	0.98	0.98	1.00	0.99	1.00	0.98	0.98	0.97	0.99
Sichuan	0.98	0.95	1.00	0.98	0.95	0.94	0.95	0.97	0.95	1.00	0.90	0.90	0.91	0.95
Guizhou	1.00	0.99	1.00	1.00	0.96	0.94	0.92	0.92	0.92	0.92	0.89	0.90	0.92	0.94
Yunnan	0.97	0.97	0.98	0.98	0.97	0.97	0.96	0.95	0.95	0.95	0.93	0.94	0.94	0.96
Shaanxi	0.90	0.90	0.91	0.91	0.91	0.90	0.90	0.90	0.90	0.89	0.86	0.86	0.86	0.89
Gansu	0.94	0.94	0.94	0.94	0.94	0.94	0.94	0.94	0.94	0.93	0.92	0.92	0.91	0.93
Qinghai	0.99	0.99	0.99	0.99	0.99	0.99	0.99	0.99	0.99	0.98	0.98	0.98	0.98	0.99
Ningxia	1.00	1.00	1.00	1.00	1.00	1.00	1.00	1.00	1.00	0.99	0.99	0.99	0.99	1.00
Xinjiang	0.97	0.96	0.96	0.96	0.96	0.97	0.96	0.96	0.95	0.95	0.93	0.91	0.92	0.95
Average	0.95	0.95	0.96	0.96	0.96	0.95	0.95	0.95	0.95	0.95	0.91	0.91	0.91	0.94

### 4.1 Decomposition of the potential number of unreasonable hospitalizations

#### 4.1.1 Input–output efficiency values that do not account for hospitalizations

This study employs the SBM-DDF model to evaluate the efficiency of primary healthcare from 2010 to 2022, excluding undesired outputs. The resulting score, $\rho_{1}$ , serves as a benchmark for comparison with $\rho_{2}$ . From an input–output perspective, $\rho_{1}\neq 1$ indicates sub-optimal production. By identifying macro-level efficiency losses reflected in inefficiency metrics, the study decomposes outputs into the number of potentially reasonable and unreasonable hospitalizations. Detailed input–output efficiency values are presented in Table 4.

#### 4.1.2 Input–Output efficiency values that account for hospitalizations

Assuming an unreasonable increase in the number of hospitalizations may lead to a decline in the overall input–output efficiency of primary healthcare institutions. This study attempts to incorporate the number of hospitalizations as an adverse output into the efficiency evaluation model and explores its impact on overall efficiency to verify whether there are unreasonable hospitalization cases. The SBM-DDF-Undersirable model is used to measure the efficiency $\rho_{2}$ of China’s primary healthcare, with specific results shown in Table 5.

**Table 5:** Input–output efficiency and its mean value (SBM-DDF-Undesirable) from 2010 to 2022.

Province	2010	2011	2012	2013	2014	2015	2016	2017	2018	2019	2020	2021	2022	Average ( $\bar{\rho_{2}^{*}}$ )
Beijing	0.99	0.99	0.99	1.00	1.00	1.00	1.00	1.00	1.00	1.00	1.00	1.00	1.00	1.00
Tianjin	0.99	1.00	0.99	1.00	1.00	1.00	1.00	1.00	1.00	1.00	1.00	1.00	1.00	1.00
Hebei	0.73	0.74	0.75	0.77	0.77	0.76	0.75	0.73	0.71	0.72	0.71	0.71	0.70	0.74
Shanxi	0.83	0.84	0.84	0.84	0.85	0.85	0.84	0.84	0.83	0.83	0.84	0.85	0.85	0.84
Inner Mongolia	0.90	0.90	0.91	0.91	0.91	0.91	0.90	0.89	0.89	0.90	0.90	0.90	0.89	0.90
Liaoning	0.86	0.86	0.86	0.86	0.87	0.87	0.87	0.86	0.86	0.87	0.87	0.86	0.86	0.86
Jilin	0.92	0.92	0.92	0.92	0.92	0.92	0.92	0.92	0.91	0.92	0.90	0.90	0.90	0.92
Heilongjiang	0.88	0.89	0.89	0.89	0.89	0.88	0.88	0.87	0.89	0.88	0.89	0.89	0.89	0.89
Shanghai	1.00	1.00	1.00	1.00	1.00	1.00	1.00	1.00	1.00	1.00	1.00	1.00	1.00	1.00
Jiangsu	0.83	0.83	0.84	0.84	0.84	0.84	0.83	0.80	0.78	0.76	0.75	0.76	0.74	0.80
Zhejiang	0.95	0.96	0.97	0.98	0.98	0.99	0.99	0.99	0.99	1.00	0.99	1.00	1.00	0.98
Anhui	0.83	0.84	0.83	0.84	0.84	0.84	0.84	0.82	0.83	0.85	0.85	0.85	0.83	0.84
Fujian	0.87	0.87	0.86	0.87	0.88	0.88	0.88	0.88	0.88	0.88	0.89	0.90	0.89	0.88
Jiangxi	0.80	0.79	0.77	0.78	0.80	0.80	0.79	0.77	0.77	0.79	0.80	0.79	0.80	0.79
Shandong	0.67	0.69	0.77	1.00	0.83	0.78	0.74	0.72	0.72	0.73	0.66	0.69	0.71	0.75
Henan	0.64	0.67	0.67	0.69	0.73	0.71	0.73	0.69	0.63	0.64	0.62	0.62	0.60	0.66
Hubei	0.80	0.80	0.78	0.77	0.77	0.75	0.73	0.72	0.70	0.70	0.70	0.71	0.70	0.74
Hunan	0.68	0.65	0.63	0.62	0.61	0.59	0.58	0.57	0.56	0.53	0.55	0.57	0.59	0.60
Guangdong	0.82	0.84	0.86	0.92	1.00	0.93	0.93	1.00	0.97	1.00	0.78	0.81	0.81	0.90
Guangxi	0.78	0.79	0.78	0.75	0.75	0.76	0.76	0.75	0.75	0.71	0.70	0.69	0.67	0.74
Hainan	0.99	0.99	0.90	0.99	0.99	0.99	0.99	0.99	0.99	0.99	0.98	0.97	0.98	0.98
Chongqing	0.86	0.86	0.84	0.83	0.83	0.82	0.82	0.81	0.81	0.80	0.80	0.80	0.80	0.82
Sichuan	0.56	0.57	0.52	0.53	0.53	0.54	0.52	0.49	0.50	0.48	0.50	0.50	0.50	0.52
Guizhou	0.83	0.83	0.80	0.80	0.82	0.83	0.83	0.83	0.82	0.81	0.81	0.81	0.80	0.82
Yunnan	0.87	0.88	0.87	0.86	0.86	0.86	0.85	0.84	0.83	0.83	0.82	0.82	0.81	0.85
Shaanxi	0.87	0.87	0.86	0.86	0.86	0.86	0.86	0.84	0.84	0.84	0.85	0.85	0.84	0.85
Gansu	0.90	0.90	0.89	0.90	0.90	0.90	0.89	0.88	0.88	0.88	0.87	0.88	0.87	0.89
Qinghai	0.99	0.98	0.98	0.98	0.98	0.98	0.98	0.98	0.98	0.98	0.98	0.98	0.98	0.98
Ningxia	1.00	1.00	1.00	1.00	1.00	1.00	1.00	1.00	1.00	1.00	1.00	1.00	1.00	1.00
Xinjiang	0.90	0.90	0.89	0.89	0.88	0.88	0.88	0.88	0.88	0.88	0.90	0.89	0.90	0.89
Average	0.85	0.85	0.85	0.86	0.86	0.86	0.85	0.85	0.84	0.84	0.83	0.83	0.83	0.85

#### 4.1.3 The severity of the potential unreasonable volume of hospitalizations

This study compares the efficiency of primary healthcare before and after treating hospitalization as an undesirable output. The results reveal divergent patterns across regions. In areas such as Beijing and Shanghai, the measured efficiency of primary healthcare increased from 0.99 to 1.00 when hospitalization services were included. This improvement indicates that inpatient care functions as an integral and value-adding component of the healthcare system in these regions, contributing to the optimization of overall resource utilization. Conversely, regions such as Henan and Hebei experienced a decline in efficiency, from 0.91 to 0.66 and from 0.82 to 0.74, respectively, after hospitalization was incorporated into the model. This decrease suggests the presence of inefficient practices or potential resource misallocation in inpatient care in these areas.

Nationwide, overall efficiency decreased by 10.1% after accounting for hospitalization as an output, highlighting the significant impact of potentially unnecessary or poorly managed hospital admissions. This finding underscores the severity of inefficient hospitalization practices at a national level and emphasizes the urgent need for targeted policy intervention and corrective measures. Detailed data are provided in Table 6.

**Table 6:** The severity of the potential unreasonable volume of hospitalizations.

Province	SBM-DDF ( $\bar{\rho_{1}^{*}}$ )	SBM-DDF-Undesirable ( $\bar{\rho_{2}^{*}}$ )	Efficiency difference ( $\bar{\rho_{1}^{*}}-\bar{\rho_{2}^{*}}$ )	Severity of unreasonable volume of hospitalizations ( $\frac{\bar{\rho_{1}^{*}}-\bar{\rho_{2}^{*}}}{\bar{\rho_{1}^{*}}}$ )
Beijing	0.99	1.00	−0.01	−1.01%
Tianjin	1.00	1.00	0.00	0.00%
Hebei	0.82	0.74	0.08	9.76%
Shanxi	0.85	0.84	0.01	1.18%
Inner Mongolia	0.92	0.90	0.02	2.17%
Liaoning	0.89	0.86	0.03	3.37%
Jilin	0.92	0.92	0.00	0.00%
Heilongjiang	0.92	0.89	0.03	3.26%
Shanghai	0.99	1.00	−0.01	−1.01%
Jiangsu	0.95	0.80	0.15	15.79%
Zhejiang	0.98	0.98	0.00	0.00%
Anhui	0.95	0.84	0.11	11.58%
Fujian	0.94	0.88	0.06	6.38%
Jiangxi	0.95	0.79	0.16	16.84%
Shandong	0.91	0.75	0.16	17.58%
Henan	0.91	0.66	0.25	27.47%
Hubei	0.95	0.74	0.21	22.11%
Hunan	0.93	0.60	0.33	35.48%
Guangdong	0.97	0.90	0.07	7.22%
Guangxi	0.95	0.74	0.21	22.11%
Hainan	0.98	0.98	0.00	0.00%
Chongqing	0.99	0.82	0.17	17.17%
Sichuan	0.95	0.52	0.43	45.26%
Guizhou	0.94	0.82	0.12	12.77%
Yunnan	0.96	0.85	0.11	11.46%
Shaanxi	0.89	0.85	0.04	4.49%
Gansu	0.93	0.89	0.04	4.30%
Qinghai	0.99	0.98	0.01	1.01%
Ningxia	1.00	1.00	0.00	0.00%
Xinjiang	0.95	0.89	0.06	6.32%
Average	0.94	0.85	0.09	10.10%

### 4.2 Static assessment of primary healthcare efficiency

In this study, the average efficiency values of primary medical institutions in each province (excluding Tibet) are calculated for 2010, 2022, and the entire 13-year period, respectively. From the perspective of average efficiency, the highest efficiency of primary health care in Tianjin and Ningxia was 1.00, while the lowest was 0.74 in Hebei province. From 2010 to 2022, the average SBM-DDF efficiency score of China’s primary healthcare institutions was 0.92. It is worth noting that the efficiency of primary medical care in different regions is uneven, and there are large regional differences. Detailed data are presented in Table 3.

**Figure 2:**
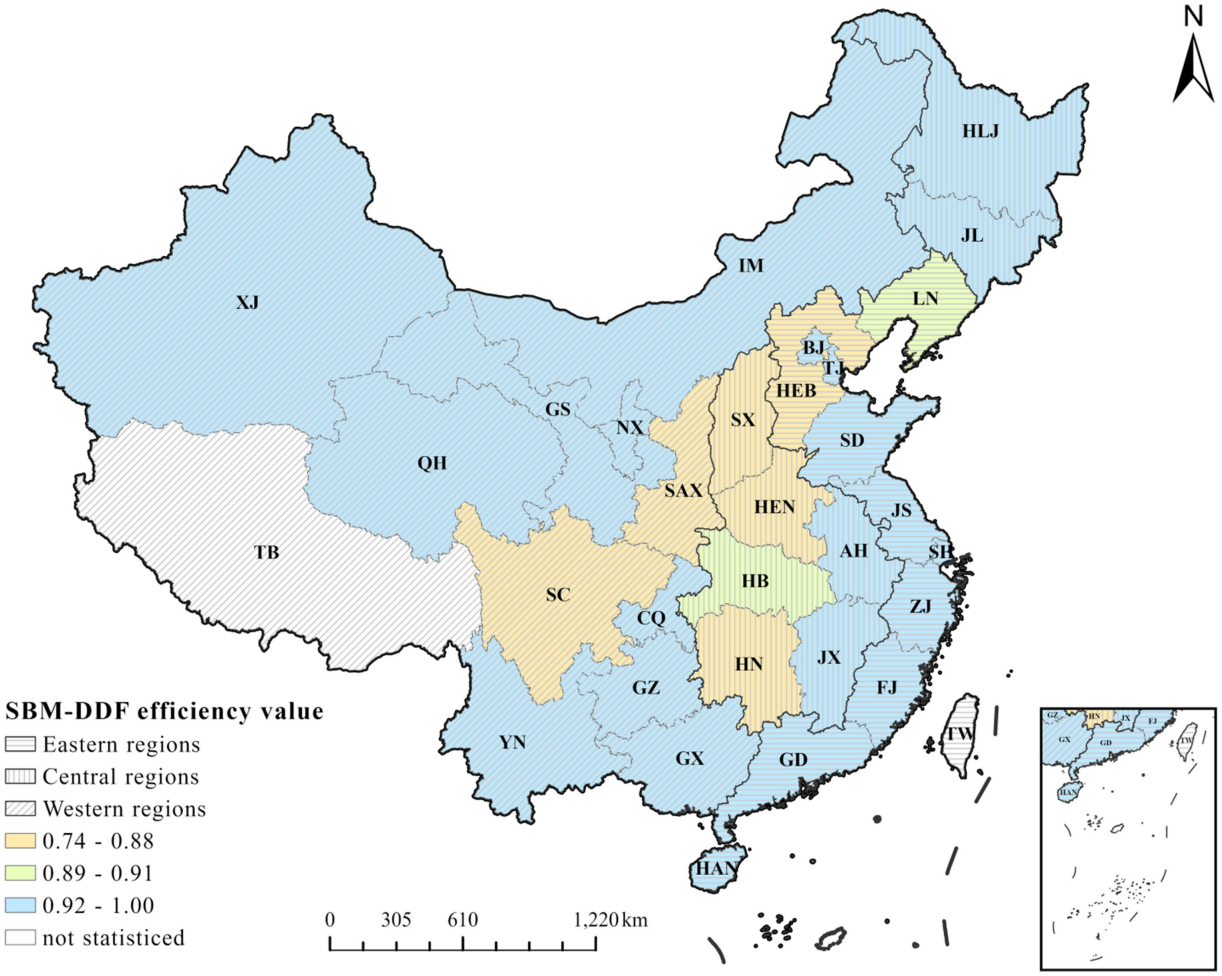
The average SBM-DDF values of primary healthcare in China from 2010 to 2022.

**Figure 3:**
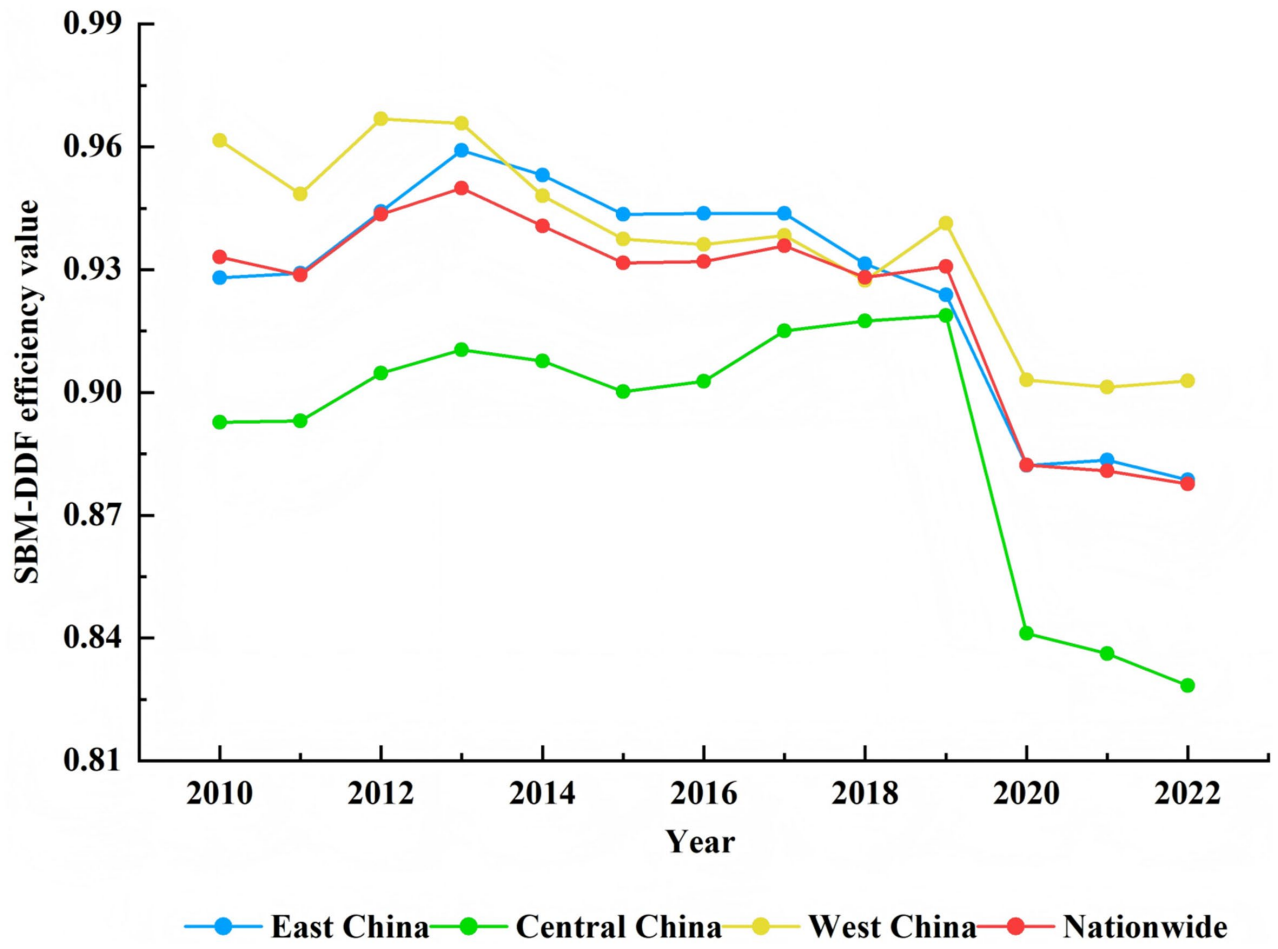
Trends of primary healthcare efficiency in eastern, central, and western China.

To further visualize the differences in the efficiency of primary health care, this paper presents comparative charts of the mean SBM-DDF efficiency for each province from 2010 to 2022. Since there are significant differences in the level of economic development between the eastern, central, and western regions of China, which affects the efficiency of medical and health resource allocation (60, 61). This paper further visualizes the efficiency values of the eastern, central and western regions over the years and studies the regional differences. See Figure 2 and Figure 3 for details.

According to Figure 2, from 2010 to 2022, regions with lower average efficiency in primary healthcare were mainly concentrated in the central region, such as Henan (0.807), while regions with higher efficiency were mainly concentrated in the eastern and western regions, such as Beijing, Tianjin, and Ningxia (0.99,1.00 and 0.998 respectively). Overall, the efficiency of primary healthcare in the eastern and western regions of China was higher than that in the central region, but the overall efficiency gap between the central region and the other two regions was relatively large.

According to Figure 3, from 2010 to 2022, the efficiency of primary healthcare services in China has experienced significant fluctuations, generally showing a downward trend. The decline was most pronounced in 2020, which may be related to the COVID-19 pandemic. Notably, the average efficiency in central regions was generally lower than the national average, reflecting a concerning trend of redundant inputs, insufficient expected outputs, or excessively high unintended outputs. The root cause of the disparity between eastern, western, and central regions lies in resource allocation that prioritizes fairness based on population rather than geographical dimensions. Therefore, distributional equity based on population and economic factors is superior to geographical factors (62–64). From a socioeconomic perspective, eastern provinces have higher gross domestic product (GDP) and better development levels (65), providing the necessary material foundation for their healthcare development. In terms of policy support, the state has recently vigorously promoted the development of western regions and continuously introduced outstanding talents in the healthcare sector, which may be potential reasons for the sustained excellence of eastern and western regions.

### 4.3 Dynamic assessment of primary healthcare efficiency

From 2010 to 2022, the GML index of total factor productivity for primary health care was calculated and decomposed for 30 provinces (autonomous regions and municipalities) in China. The GML values were greater than 1, indicating that the total factor productivity of primary healthcare increased compared to the previous year during this initial period. In contrast, the GML values were less than 1, suggesting a downward trend in total factor productivity during these years. And we found the GML index of total factor productivity for primary healthcare in China from 2010 to 2022 exhibited a fluctuating trend. The specific data are detailed in Table 7.

**Table 7:** GML index of total factor productivity of primary health care in China from 2010 to 2021.

Region	Year											
	2010/2011	2011/2012	2012/2013	2013/2014	2014/2015	2015/2016	2016/2017	2017/2018	2018/2019	2019/2020	2020/2021	2021/2022
Beijing	1.00	1.00	1.01	1.00	1.00	1.00	1.00	1.00	1.00	0.98	1.01	1.00
Tianjin	1.00	1.00	1.00	1.00	1.00	1.00	1.00	1.00	1.00	1.00	1.00	1.00
Hebei	1.01	1.02	1.02	1.00	1.00	0.99	0.97	0.97	0.99	0.99	1.00	0.98
Shanxi	1.00	1.00	1.00	1.00	1.00	1.00	1.00	1.00	1.00	1.00	1.00	1.00
Inner Mongolia	1.00	1.00	1.00	1.00	1.00	1.00	1.00	1.00	1.00	1.00	1.00	1.00
Liaoning	1.00	1.00	1.00	1.00	1.00	1.00	1.00	0.99	1.00	0.99	1.00	1.00
Jilin	1.00	1.00	1.00	1.00	1.00	1.00	1.00	0.99	1.00	0.99	1.00	1.00
Heilongjiang	0.99	1.00	1.00	1.00	1.00	1.00	1.00	0.99	0.99	1.00	1.00	1.00
Shanghai	1.00	1.00	1.00	1.00	1.00	1.00	1.00	1.00	1.00	0.99	1.00	0.99
Jiangsu	1.00	1.01	1.02	1.01	1.01	0.99	1.04	0.96	0.98	0.92	1.00	0.97
Zhejiang	1.01	1.01	1.00	1.00	1.00	1.00	1.00	1.00	1.01	0.97	1.01	1.02
Anhui	1.00	1.03	1.02	1.00	0.99	0.99	1.00	0.99	0.99	0.97	0.99	0.98
Fujian	1.00	1.00	0.99	0.99	1.00	1.00	1.00	1.00	1.00	0.99	1.00	0.99
Jiangxi	1.00	0.94	1.01	0.98	0.99	1.00	1.01	0.99	0.99	0.97	0.99	1.00
Shandong	1.02	1.19	1.00	0.93	0.92	1.01	1.00	0.97	0.95	0.94	1.02	1.01
Henan	1.00	1.05	0.97	1.03	0.99	1.01	1.03	0.96	0.98	0.91	0.98	0.96
Hubei	1.00	1.04	1.00	1.00	0.99	1.01	1.07	0.93	1.07	0.84	1.01	0.99
Hunan	1.01	1.02	1.03	0.97	0.99	1.01	1.00	1.18	1.00	0.81	1.00	1.02
Guangdong	0.98	1.02	1.03	1.00	0.99	1.01	1.00	0.99	1.01	0.86	1.00	0.99
Guangxi	0.97	1.02	1.04	0.96	0.98	0.99	0.99	0.99	1.01	0.96	0.99	1.00
Hainan	1.00	0.92	1.09	1.00	1.00	1.00	1.00	1.00	1.00	0.99	1.00	1.00
Chongqing	1.00	1.03	1.00	0.99	0.98	1.00	1.03	0.98	1.03	0.96	1.01	0.99
Sichuan	0.95	1.13	0.94	0.94	0.97	1.01	1.04	0.96	1.15	0.79	0.99	1.02
Guizhou	0.98	1.02	0.99	0.95	0.98	0.99	0.99	1.00	1.00	0.98	1.01	1.01
Yunnan	0.99	1.01	1.00	0.99	0.99	1.00	0.98	0.99	1.00	0.98	1.00	1.00
Shaanxi	1.00	1.00	1.00	1.00	1.00	1.00	1.00	1.00	0.99	0.99	1.00	1.00
Gansu	1.00	1.00	1.01	1.00	1.00	1.00	1.00	1.00	1.00	0.99	1.00	0.99
Qinghai	1.00	1.00	1.00	1.00	1.00	1.00	1.00	1.00	1.00	1.00	1.00	1.00
Ningxia	1.00	1.00	1.00	1.00	1.00	1.00	1.00	1.00	1.00	1.00	1.00	1.00
Xinjiang	0.99	1.00	1.00	1.00	1.00	1.00	0.99	0.99	1.00	0.99	0.99	1.01
Average	1.00	1.02	1.01	0.99	0.99	1.00	1.00	0.99	1.00	0.96	1.00	1.00

**Figure 4:**
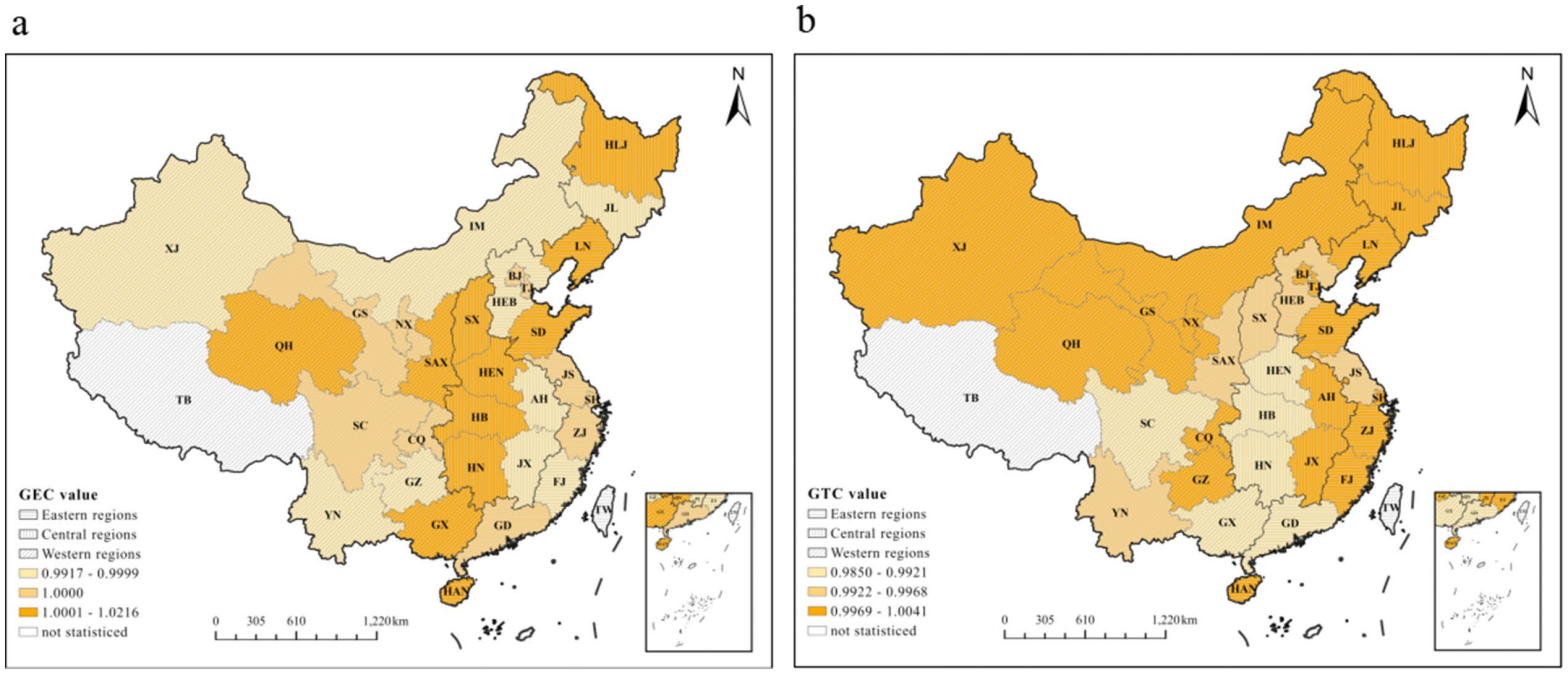
The average GEC and GTC values of primary healthcare in China from 2010 to 2022.

**Figure 5:**
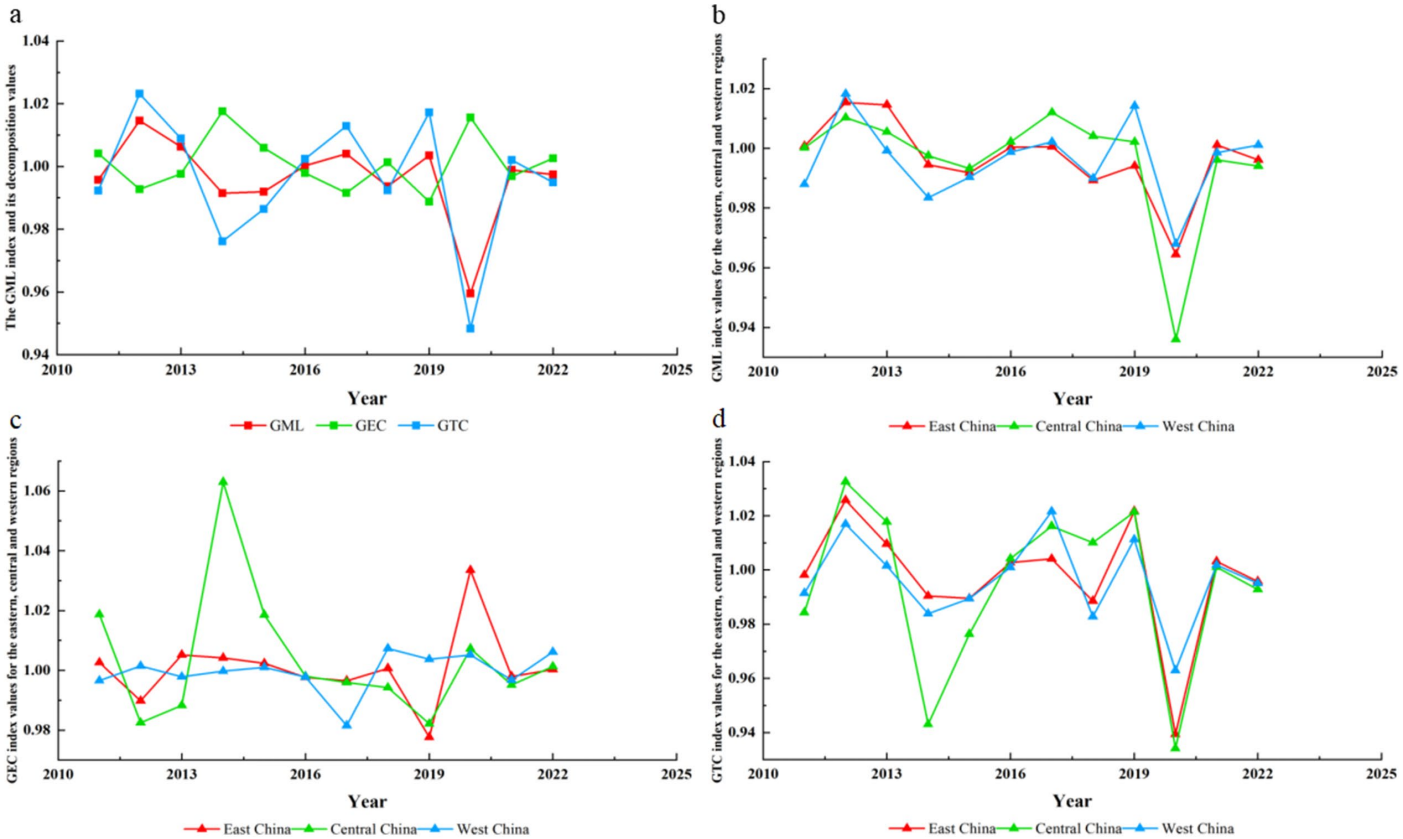
Dynamic evaluation of primary healthcare efficiency. **(a)** The GML index and its decomposition. **(b)** GML index values for the eastern, central, and western regions. **(c)** GEC index values for the eastern, central, and western regions. **(d)** GTC index values for the eastern, central, and western regions.

To further explore the characteristics of Global Efficiency Change (GEC) and Global Technological Change (GTC) indices under the decomposition of the national primary medical and health GML index, as well as the differences between regions, we calculated the GML index and its decomposition indices for all primary medical and health care production factors across 30 provinces (autonomous regions and municipalities) in China. Additionally, we created a comparison chart of the GEC and GTC indices in the eastern, central and western regions from 2010 to 2022. It is worth noting that rounding the values in Figure 4‘s legend to two decimal places would obscure the subtle differences critical to interpreting the spatiotemporal evolution of efficiency, hence four decimal places are retained here. These results are presented in Figure 5 and Figure 4.

According to Figure 5, (a) between 2010 and 2022, China’s GML index and its decomposition indicators in primary healthcare demonstrated significant fluctuations and instability. Moreover, changes in GML values were notably influenced by GEC and GTC. When GML exceeded 1, it showed an upward trend accompanied by high GTC values, indicating that advancements in medical technology may enhance healthcare efficiency. (b) Compared to eastern and western regions, central China exhibited greater volatility in GML index fluctuations, suggesting highly unstable efficiency development trends that require attention. Notably, when decomposition indicators were simultaneously positive or negative, GML index changes became particularly pronounced. This indicates that synchronized improvements in technical and managerial capabilities at primary healthcare institutions would lead to more significant health efficiency gains. (c) The average GEC index in eastern, central, and western regions showed substantial fluctuations, with central China demonstrating particularly notable instability. This reflects inconsistent effectiveness in management systems and resource allocation mechanisms for primary healthcare institutions in central China, necessitating further exploration of long-term stable institutional frameworks. (d) The GTC index in eastern, central, and western regions all experienced significant declines in 2020, likely due to the impact of the COVID-19 pandemic that compromised the utility of primary healthcare technologies (such as outpatient services and clinical surgical skills). This highlights the need to prioritize technological development during emergency situations.

According to Figure 4: (a) Regarding the Technical Efficiency Change Index (GEC), the average GEC value of primary healthcare in most eastern and western regions exceeded 1 between 2010 and 2022. This indicates sustained optimization trends in healthcare management systems and resource allocation mechanisms across these regions, likely driven by national policy support or local institutional development. In contrast, most central regions recorded GEC values below 1, reflecting inefficiencies or irrational resource distribution in their primary healthcare systems. (b) For the Technical Progress Change Index (GTC), the average efficiency value of primary healthcare institutions in most regions remained below 1 during the same period. This suggests significant room for improvement in outpatient technical skills and clinical surgical capabilities, with the distribution of disparities mirroring the pattern observed in the left-hand GEC graph.

### 4.4 Forecast analysis of primary healthcare demand

To research the development trend of diagnosis and treatment demand at primary township medical and health centers over the next few years, we built a single time-series model with Random Forest (RF). The analysis of the forecast results shows that the outpatient volume in 2029 will reach about 1.07 billion, and the overall trend is rising, which provides a certain theoretical basis for promoting the preemptive allocation of medical resources. Meanwhile, this study compared RF with Support Vector Regression (SVR), Lasso Regression, and Linear Regression to demonstrate the advantages of it in prediction tasks. Although performance metrics on the training set reflect the model’s fitting degree to observed data, such evaluation carries the risk of overfitting and cannot objectively demonstrate the model’s generalizability to unseen data in real-world scenarios. Therefore, this study further employs an independent test set for validation, utilizing Mean Absolute Error (MAE), Mean Absolute Percentage Error (MAPE), Mean Squared Error (MSE), and Root Mean Squared Error (RMSE) as core evaluation metrics. The detailed results are presented in Table 8.

**Table 8:** Comparison of predictive model performance on the test set (2019–2022).

Methods	MAE	MAPE	MSE	RMSE
Random forest	0.54	4.61	0.37	0.61
SVR	1.61	13.73	3.16	1.78
Lasso regression	2.17	18.67	4.88	2.21
Linear regression	2.17	18.67	4.88	2.21

According to Table 8, The random forest model performs relatively well on the test set, with MAE, MAPE, MSE, and RMSE values of 0.54275,4.610215,0.368475, and 0.607022, respectively. Compared to typical models like SVR, Lasso Regression, and Linear Regression, the RF model demonstrates certain predictive advantages, which also validates the rationality of its selection.

## 5 Discussion

The inter-regional gap and spatiotemporal heterogeneity in the efficiency of primary medical services reflect the existing contradictions in the supply structure of medical resources in China. In this study, we construct the SBM-DDF model to quantitatively evaluate the efficiency of primary care resource allocation, taking into account undesirable outputs. Additionally, the GML index is used to analyze the trends in primary health care efficiency and its components. Furthermore, this study predicts the number of primary medical visits, providing a theoretical basis for the rational allocation of primary medical resources.

### 5.1 The connections and differences of static and dynamic assessments

Static and dynamic evaluations are complementary yet distinct. Specifically, the static SBM-DDF model forms the basis of the dynamic GML index. The GML index and its decomposed components directly measure changes in technological efficiency relative to the global frontier, which is derived from intertemporal SBM-DDF results. However, the research directions and objectives of static and dynamic approaches differ. Static assessment focuses on efficiency levels at specific time points, while dynamic assessment tracks efficiency changes over time. Static evaluation primarily diagnoses current resource allocation and identifies structural inefficiencies in specific years. Dynamic evaluation, on the other hand, tracks trends and breaks down productivity growth drivers to distinguish between improvements from management optimization (GEC) and technological progress (GTC). In general, it is important to clarify the differences and connections between static and dynamic assessments, which will help to better leverage their roles.

### 5.2 The divergent impact of hospitalizations on efficiency

This study identifies macro-level efficiency losses at suboptimal levels, decomposing total hospitalizations into potential number of reasonable and unreasonable hospitalizations while examining the severity and regional disparities of unreasonable hospitalization. Findings reveal declining input–output efficiency in regions like Henan and Hebei, suggesting issues such as unreasonable hospitalization practices or resource waste. From a social benefit perspective, although increasing hospitalization admissions generates revenue for healthcare institutions, indiscriminate expansion of unnecessary medical services hinders rational allocation of primary healthcare resources. In contrast, cities like Beijing and Shanghai demonstrate improved hospitalization service efficiency, indicating that hospitalization plays a necessary and rational role in these areas, contributing positively to healthcare output. By analyzing the severity of irrational hospitalizations, this study effectively identifies regional disparities in hospitalization impacts, providing theoretical foundations for policymakers.

### 5.3 Regional disparities in primary healthcare efficiency

The study reveals that primary health care efficiency in eastern China was consistently higher than in western regions from 2010 to 2022, highlighting a pronounced disparity of “high efficiency in the east and west versus low efficiency in the central region.” Notably, the central region’s average efficiency consistently fell below the national average, indicating concerning trends of resource redundancy, underutilized potential, or excessive unintended outputs. When evaluating distributional equity based on demographic and economic factors (62–64), the central region demonstrated greater advantages than geographical factors. By driving economic development to boost healthcare services and actively recruiting medical professionals, the central region has established essential material foundations for healthcare advancement, thereby mitigating disparities in primary health care delivery.

### 5.4 Underlying mechanisms of regional imbalances

In order to further explore the causes of regional imbalance, we can consider different factors, such as differences in public funding, differences in health infrastructure, the availability of qualified personnel and urban–rural inequalities. Firstly, in terms of public funds, the eastern, central and western regions may be affected by the differences in medical service objects or the roles they play in medical services. The different government medical expenditures affect the input under the efficiency system, which is an important factor leading to the differences between the eastern, central and western regions. Secondly, in terms of health infrastructure, medical capital investment may affect the efficiency of primary healthcare in the different regions. However, the economic development in the eastern region is higher than that in other regions, and the speed of facility renewal may be faster, which is an important factor for the continuous advantage of medical efficiency in the eastern region. Thirdly, in terms of the level and efficiency of medical services, if the level of medical services is better, it may be favored by more patients, which is conducive to the full utilization of resources. Therefore, the efficiency difference brought by medical services may be one of the important reasons for the imbalance between eastern, central and western regions. Fourthly, in terms of the availability of qualified personnel, if primary healthcare institutions recruit a large number of medical workers but cannot make full use of them, it may cause a waste of human resources. That is to say, the availability of qualified personnel may be the main reason for the difference in efficiency of primary medical and health care. Finally, when exploring the reasons for the imbalance of medical efficiency between eastern, central and western regions, the development difference between urban and rural areas is an important factor that cannot be ignored, which may affect patients’ preference for primary hospitals and large medical institutions. In summary, a detailed investigation into the root causes of these imbalances may help improve the policy relevance and explanatory power of the research.

### 5.5 Prediction of future primary care demand

Through the prediction of primary care demand, it is found that the number of visits to primary medical institutions will show an overall upward trend from 2026 to 2029. This finding differs from Luo’s prediction of the number of daily outpatient visits in hospitals (16). While short-term predictions can capture the impact of emergencies on medical visits, this study focuses more on predicting the annual number of patients from a macro perspective. This approach aims to provide a theoretical basis for strengthening regional cooperation and achieving flexible scheduling of medical resources.

### 5.6 Policy recommendations

Similarly, it is important to note that the overall efficiency of primary medical resource allocation is lower than that of higher-level medical institutions. This may be attributed to the loss of resources due to residents’ preference for large hospitals in their healthcare choices and a lack of trust in primary medical and health institutions (58, 66). Based on the research results, this paper tries to put forward specific and feasible suggestions and strategies for improvement. First of all, there is a large space for improving the efficiency of medical resource allocation in central and western regions. The government could focus on building a regional cooperation framework to promote the coordinated development of regional medical resource allocation capacity. Secondly, the comprehensive medical reform pilot plays an important role in improving the efficiency of medical resource allocation. Policymakers can encourage the in-depth development of comprehensive medical reform, strengthening regional cooperation and promoting resource sharing through medical alliances and integrated healthcare communities (67). Thirdly, the allocation of medical resources should be optimized by the market. Establishing an effective market is crucial to eliminating the imbalance of resource allocation.

### 5.7 Limitations and future directions

This study has several limitations that should be noted: First, due to the lag in the informatization construction of primary medical and health institutions, the time span of the relevant datasets included in this study is relatively short. This limits the comprehensiveness of the analysis. Second, the selection of input–output indicators is subject to some degree of subjectivity and could benefit from a more systematic approach to ensure robustness and generalizability. Third, with only 33 annual observations and a single sequential hold-out split, our medical-demand forecast lacks the temporal depth needed for time-series cross-validation (TSCV). This constraint prevents a thorough out-of-sample evaluation, so the projected 1.07 billion outpatient visits for 2029 is best viewed as a tentative benchmark that should be interpreted with caution.

Future research can be enhanced by exploring several key directions: Firstly, a more rigorous method should be adopted for selecting evaluation indicators, ensuring effective differentiation between input and output indicators. Secondly, the development of a hybrid evaluation framework that integrates machine learning with non-parametric models, such as DEA-Transformer, could better track dynamic efficiency and align evaluation results more closely with real-world conditions. Thirdly, longer time-series data and robust validation techniques such as time-series cross-validation (TSCV) can be used to predict future primary-care visits, capturing subtler changes in medical demand and improving forecast accuracy.

Regarding multi-year map variations, this study currently focuses on the analysis of average efficiency and overall productivity change indices. However, due to different research perspectives, there is a possibility of multi-year efficiency map variations. Specifically, this study provides a macro-level assessment, incorporating numerous primary healthcare institutions into an input–output system under the strong assumption of ignoring structural differences in case structures. From a macro-management perspective, it analyzes the regional differences in the overall average efficiency and dynamic trends of overall productivity in China’s primary health care efficiency from 2010 to 2022. Future research could focus on developing year-by-year or phased spatial evolution maps to visualize changes in primary health care efficiency across China’s regions. Through a forward-looking perspective, this approach would dynamically reveal geographical patterns and spatiotemporal pathways of efficiency evolution, moving to track regional shifts over time. Furthermore, building on the year-by-year or phased spatial evolution maps, we could integrate case-mix characteristics and local technical resources to enrich the visualization system with region-specific determinants. This would help identify key stages, turning points, and influencing factors in the efficiency evolution of different regions, thereby providing a scientific basis for differentiated and targeted health policy formulation. This research direction holds significant theoretical innovation and policy application value, serving as one of the key pathways to enhance the insight and foresight of primary health care evaluation systems.

## 6 Conclusion

This study scientifically quantifies excessive medical care in primary-level institutions and incorporates the undesirable output into the evaluation system. By integrating a comprehensive method based on slacks-based measure, directional distance function and global Malmquist-Luenberger (SBM-DDF-GML), we assess both static and dynamic efficiency of China’s primary-care providers from 2010 to 2022. The research reveal widespread overtreatment and large regional disparities in resource-allocation efficiency, with the greatest improvement potential in central and western provinces. These findings hope to offer policymakers actionable evidence for reallocating healthcare resources more rationally and efficiently.

## Data Availability

The datasets presented in this study can be found in online repositories. The names of the repository/repositories and accession number(s) can be found at: https://www.nhc.gov.cn/mohwsbwstjxxzx/tjtjnj/202501/8193a8edda0f49df80eb5a8ef5e2547c.shtml.
